# Treatment of Eczema

**Published:** 1885-06

**Authors:** Henry J. Reynolds

**Affiliations:** Professor of Dermatology in the College of Physicians and Surgeons, Chicago. Dermatologist to the West Side Free Dispensary, Chicago. Surgeon to the Department of Genito-Urinary Diseases, West Side Free Dispensary, Chicago; 2200 Michigan Ave.


### ARTICLE III.

**TREATMENT OF ECZEMA.** *By* HENRY J. REYNOLDS, M. D.,
*Professor of Dermatology in the College of Physicians and Surgeons, Chicago. Dermatologist to the West Side Free Dispensary, Chicago. Surgeon to the Department of Genito-Urinary Diseases, West Side Free Dispensary, Chicago.*

[Read before the Illinois State Medical Society, May 21, 1885.]

As this subject is a very broad one, and one, in order to treat which fully, would occupy too much time, my remarks will be directed more particularly to the principles necessarily involved in the same; an intelligent knowledge of which always suggests the particular remedy or form of treatment that will be applicable in each case as it is encountered. This fact will be the more fully realized when we remember that upwards of one hundred names have been made use of by the various authors to designate different forms of the disease. Brief reference will also be made to a few remedies which I have myself found to be useful in certain cases.

I shall assume, as experience has led me to believe, that while there may be constitutional predisposing causes for the disease, the direct or exciting cause is usually external; certain it is, however, that many forms may be produced by external irritation, and that all may be aggravated by it. A consideration of these facts suggests the idea that the treatment, to be effectual, must be, to a great extent, external or local in character; and again, as many cases tend to spontaneous recovery, when the part has been simply protected from external irritation, for example, the eczema intertrigo, or that produced by filth and chafing, that resulting from parasitic irritation, the application of poisonous substances to the skin, etc., the rational inference is, that the external treatment must be directed with a view to removing all irritation of a local or external character.

In a disease which assumes so many different forms, no specific line of treatment or rule can be laid down that will be applicable to all cases; the remedies must be adapted to the particular existing conditions met with in each case. What would prove useful in one stage, in one form, in one locality or in one person, may be not only useless in another, but absolutely injurious. . The secret of successful management in this disease, more than many others, perhaps, lies not so much in the possession of an unlimited knowledge of remedies as it does in the display of common sense and good judgment in the adaptation of remedies to conditions met with. The pathological condition being absolutely identical in no two cases, so the treatment must vary, and a knowledge of specified lines of treatment or certain combinations of drugs said to be useful, with a neglect of consideration of the principles upon which treatment should be based in each individual case, in this, as in all other diseases, is liable to mislead.

With these few ideas constantly in mind, with the confidence and coöperation of the patient, with the possession of good judgment and common sense, with even a limited amount of scientific knowledge, the physician is prepared to intelligently undertake the management of each case as it presents itself, no matter what its particular form or name may be, and in the majority of cases, under such circumstances, is warranted in guaranteeing a cure, though in a disease which, untreated, naturally tends to chronicity, it is of the utmost importance that in rendering a prognosis the physician secure for himself an abundance of time in which to complete the cure; the cooperation of the patient with regard to the length of time he is willing to persist in carrying out the prescribed treatment is as essential to the ultimate success of the same as the remedies made use of. The physician is many times defeated in a cure, in chronic cases, solely by lack of persistence in the treatment on the part of the patient.

For the purpose of convenience I think all cases presented for treatment, regardless of the name, location, or cause, may be intelligently managed by dividing them into three classes: First, those the features of which are mainly of an *acute* character; second, those forms wherein the predominating features are *sub-acute* in type; and third, that class of cases that take on the indolent or *chronic* form. The treatment may then be arranged accordingly, always subject, however, to constant variation according to conditions warranting the same; for instance, the acute may very soon take on a sub-acute form; the sub-acute may approximate in character the acute or chronic, and the chronic may at any time, owing to circumstances perhaps not under the control of the physician, develop acute symptoms. Here, as before stated, the physician is called upon for common sense, good judgment, power of discrimination, etc., which are more essential elements in successful treatment than an unlimited knowledge of remedies.

It is necessary, first, then, before adopting any line of treatment, to determine what the predominating features are: whether they be acute, sub-acute, or chronic; which, to a naturally close observer, with even a limited amount of experience, is a matter of no great difficulty.

*Acute.*—When the disease assumes this character, as in all other acute inflammatory affections, the great principle necessarily involved in successful management, is *rest;* and this does not necessarily simply imply quietude of the organ or member but *rest from every irritating influence;* whether it be motion, friction, scratching, parasitic, or atmospheric irritation, too frequent washing the dirt naturally incident to the calling of the individual, etc. To accomplish this, soothing and protecting measures are called for, among which may be mentioned equal parts of sweet almond, linseed or olive oil and lime-water. Various poultices answer as well or better in some instances, which, to be most soothing, require to be at least as warm as the temperature of the body. To either of these preparations a little laudanum may be added, if thought advisable, and to avoid too frequent exposure of the part in changing the poultice should be covered with oiled silk. With this poulticing, soothing treatment, the part will invariably pass rapidly into the sub-acute condition, when the treatment may vary accordingly.

*Sub-acute.*—In this class of cases, which may assume this form from the beginning, or may follow the acute, the part should first, if necessary, have oleaginous remedies applied three times a day for one or two days, for the purpose of loosening up dried crusts and scales. It should then be carefully washed with soap and warm water till well cleansed. I think, as we now find the condition, the various dusting powders answer the best purpose, and it is my almost universal custom, in both dispensary and private practice, to use impalpably fine boracic acid for this purpose. This powder seems to work particularly well if there be a tendency to pus formation, drying it up in a very few days invariably. Scratching, in this, as in all other forms, must be imperatively prohibited: no treatment will be effectual if the part be kept irritated by scratching, and the mildest sub-acute or chronic form can at once be aggravated into an acute condition thereby; besides, it not only fails to relieve the itching, but invariably aggravates it. A little camphor, finely pulverized and added to the boracic acid, sometimes has a tendency to relieve the itching. In my experience, however, there is nothing will prove so effectual in relieving itching as *total abstinence from scratching*, and to this end it will frequently be found necessary to either muffle or tie the hands of infants and children. I have frequently had good results from the use of a mixture of equal parts of tinct. camphoræ tinct. myrrhæ and tinct. opii, which will be well tolerated unless there be too much abrasion. The part may be washed once a day or every second day with warm water and soap, which should, in this stage, be only mildly alkaline, some of the hard or soda soaps being the preferable form. It must be borne in mind, again, however, that the symptoms may now at any time assume either the acute or chronic form, and thereby necessitate a variation in the treatment accordingly.

*Chronic.*—In this form, which may be chronic from the commencement, or may result from the sub-acute, and in which the predominating features are frequently more the results of eczema, as infiltration, thickening, hypertrophy, etc., than true eczema itself, more active and stimulating measures are required. The soaps, which, in this stage, are used for their stimulating and anti-pruritic effect, dissolving and getting rid of pathological products, which create irritation, induce, prevent physiological action, etc., should be of the strongly alkaline or potash variety, and should be freely used. After thoroughly cleansing, I have frequently had good results from the local application of the ordinary soap liniment, the camphor therein contained acting as an anti-pruritic. The different tarry preparations are very useful, especially in the squamous varieties. In chronic eczema of the leg, with which is frequently associated ulceration and varicose veins—which is frequently chronic only, however, as regards duration, the actual symptoms being often acute—the bandage is always very useful. It, in addition to enforcing the principle of *rest*

from the mechanical irritation caused by the over-loaded veins and capillaries, induces, by its contact with the tissues, absorption of morbid products and allows the parts to regain their tone.

The two following cases, which were treated at the West Side Free Dispensary and made the subjects of clinical lectures by me at the College of Physicians and Surgeons, of Chicago, may be of some interest in this connection:

CASE I. Mr. C., aged seventy-two, came under my observation in October, 1873. The right leg, from knee down, eczematous and discharging pus freely. Ulcer, just above external malleolus, four inches in diameter, also smaller one just above internal malleolus. Leg enormously enlarged and verrucous from hypertrophy and about the same size from knee to instep. Varicose veins. Patient unable to get around without crutches. This condition had existed with varying severity for twenty years and was, in short, so bad that a diagnosis of elephantiasis had previously been made and amputation advised.

The whole leg, ulcer and all, was thoroughly washed with hot water and green soap every day.

When gently wiped dry, it was sprinkled with pure boracic acid, covered with loose cloths, and bandaged with cotton roller. Scratching strictly prohibited and saline laxative ordered to be taken before each meal. This constituted the entire treatment, except that later, when there was less exudation and maceration of the tissues, the rubber bandage was substitued for the cotton, and still later an elastic stocking was worn.

Improvement commenced immediately and continued till ulcers were all healed and hypertrophy almost entirely gone. In six months' time patient was practically well and able to go without the crutches. Is still under observation and all right except that he wears the elastic stocking.

*Case II.* P. S., aged forty-eight, has general reddened, angry, moist—from exudation—condition of the entire circumference of the lower two-thirds of right leg. Also considerable swelling, intense itching at times, and an ulcer just internal to the crest of the tibia in the middle of the lower third an inch in diameter. Has also varicose veins. Has had trouble of a similar kind for twelve years and has been treated by numerous physicians, with little or no success, all of whom pronounced the trouble syphilitic.

Although there were some suspicious circumstances, examination failed to reveal satisfactory evidence of syphilitic infection.

In the treatment no anti-syphilitic medication was employed but patient treated as in "CASE I." Cure was complete in three months.

Will say, in this connection, that I regard the cotton bandage as much superior to the rubber where there is much exudation, as it allows evaporation to take place. In conclusion, I will refer briefly to the constitutional treatment.

As conditions which predispose to it necessarily aggravate or prolong the disease, it is essential that the constitutional predisposing condition be at least corrected, or that the general system be kept in a condition that will necessarily favor the desired reparative process. I think in the majority of such cases we find a faulty digestion; by the imperfect performance of which function and the consequent taking up by the lacteals of material, which instead of being nutritious and purifying to the blood acts as a poision to it, I think it is fair to presume all diseases are influenced, and many, apparently remote affections, solely depend. I consider it, therefore, very essential that the alimentary canal receive due consideration.

And again, in many eczematous subjects with ruddy complexion and apparently the picture of health, we may have a good digestion and assimilation but an imperfect *elimination* and hence, of course, a vascular fullness and consequent hyperæmia or congestion of the cutaneous capillaries that may explain the rosy complexion; a condition which only requires the slightest external irritation to kindle an acute eczema. This condition can invariably be overcome by the continued use in small doses before each meal of a saline laxative, of which there is none better than sulphate of magnesium; which, together with reducing vascular fullness and consequent cutaneous hyperæmia, very much favors digestion. In short, measures are always indicated which tend to establish and maintain harmony in the performance of the functions of the various organs of the body. Arsenic, which has always been so popular a remedy in skin affections, I very rarely use; I think in many cases it does harm. Chrysarobin, internally, which has recently been so highly recommended by Stocquart, I have tried without any visible benefit.

2200 Michigan Ave.

